# N^6^-Adenosine methylation on mRNA is recognized by YTH2 domain protein of human malaria parasite *Plasmodium falciparum*

**DOI:** 10.1186/s13072-020-00355-7

**Published:** 2020-08-31

**Authors:** Gayathri Govindaraju, Rajashekar Varma Kadumuri, Devadathan Valiyamangalath Sethumadhavan, C. A. Jabeena, Sreenivas Chavali, Arumugam Rajavelu

**Affiliations:** 1 Pathogen Biology, Rajiv Gandhi Centre for Biotechnology (RGCB), Thycaud PO, Thiruvananthapuram, Kerala 695014 India; 2 Department of Biology, Indian Institute of Science Education and Research (IISER) Tirupati, Karakambadi Road, Tirupati, Andhra Pradesh 517507 India; 3 Manipal Academy of Higher Education, Tiger Circle Road, Madhav Nagar, Manipal, Karnataka 576104 India

**Keywords:** RNA methylation, N^6^-adenosine methylation, Methyl reading, Post-transcriptional regulation, Plasmodium, Epigenetics

## Abstract

**Background:**

*Plasmodium falciparum* exhibits high translational plasticity during its development in RBCs, yet the regulation at the post-transcriptional level is not well understood. The N^6^-methyl adenosine (m6A) is an important epigenetic modification primarily present on mRNA that controls the levels of transcripts and efficiency of translation in eukaryotes. Recently, the dynamics of m6A on mRNAs at all three developmental stages of *P. falciparum* in RBCs have been profiled; however, the proteins that regulate the m6A containing mRNAs in the parasites are unknown.

**Results:**

Using sequence analysis, we computationally identified that the *P. falciparum* genome encodes two putative YTH (YT521-B Homology) domain-containing proteins, which could potentially bind to m6A containing mRNA. We developed a modified methylated RNA immunoprecipitation (MeRIP) assay using PfYTH2 and find that it binds selectively to m6A containing transcripts. The PfYTH2 has a conserved aromatic amino acid cage that forms the methyl-binding pocket. Through site-directed mutagenesis experiments and molecular dynamics simulations, we show that F98 residue is important for m6A binding on mRNA. Fluorescence depolarization assay confirmed that PfYTH2 binds to methylated RNA oligos with high affinity. Further, MeRIP sequencing data revealed that PfYTH2 has more permissive sequence specificity on target m6A containing mRNA than other known eukaryotic YTH proteins. Taken together, here we identify and characterize PfYTH2 as the major protein that could regulate m6A containing transcripts in *P. falciparum*.

**Conclusion:**

*Plasmodium* spp. lost the canonical m6A-specific demethylases in their genomes, however, the YTH domain-containing proteins seem to be retained. This study presents a possibility that the YTH proteins are involved in post-transcriptional control in *P. falciparum*, and might orchestrate the translation of mRNA in various developmental stages of *P. falciparum*. This is perhaps the first characterization of the methyl-reading function of YTH protein in any parasites.

## Background

Malaria is a major vector-borne disease predominantly affecting tropical countries. Of great concern is the emergence of drug-resistant *P. falciparum* strains that have developed resistance to all existing drug therapies, including artemisinin combination therapy [1, 2]. *Plasmodium* parasites undergo various developmental stages in the human host. Comparative transcriptomic and proteomic studies of different *P. falciparum* stages have shown that there is a marked delay between mRNA synthesis and protein expression for nearly 30% of the 2584 analyzed genes [3]. Importantly, developmental switches in *P. falciparum* are marked by broad changes in transcriptional activity [4–6]. These findings suggest that *Plasmodium* spp. have distinct regulatory mechanisms, both at the post-transcriptional and translational levels that regulate various developmental stages of the *Plasmodium* lifecycle. Such unique mechanisms to fine-tune the global gene expression might help *P. falciparum* to establish successful pathogenesis inside the human host.

Epigenetic modifications such as DNA and histone-methylation play a pivotal role in regulating gene expression and safeguarding genome integrity. Nevertheless, the knowledge of the nucleic acid methylome in human malaria parasite is limited. Discovery of reversible adenine methylation on mRNA in many higher eukaryotes has opened new avenues to explore the post-transcriptional gene regulation in eukaryotic pathogens [7, 8]. Interestingly, some apicomplexan parasites have lost DNA methylation machinery, but retain the cytosine-5 methyltransferase that methylate tRNA rather than DNA [9–11]. However, residual levels of DNA methylation have been detected in the genome of *P. falciparum* [12]. In addition to this, the number of transcription-associated proteins, such as specific transcription factors and the subunits of the mediator complex, are fewer in *P. falciparum* and *P. vivax*, as compared to other eukaryotes [13–16].

Presence of only a few transcription factors suggests that parasite might have a unique modes of gene regulation at the RNA level [17, 18]. The mRNA of *P. falciparum* may undergo stringent regulation to facilitate delayed protein synthesis in a stage-dependent manner. The stability and fine-tuning of mRNA processing could happen at two levels: (i) addition of poly (A) tails at 3′ end of RNA and/or (ii) epigenetic modifications of mRNA, particularly N^6^-adenosine methylation of the matured mRNA. RNA modifications have emerged as critical biological regulators of tRNA, rRNA, and mRNA homeostasis in eukaryotes [19]. For instance, in eukaryotes the C38 modification of tRNA aspartic acid regulates the stability of tRNA [20], whereas in *P. falciparum* tRNA methylation may have a significant role in the regulation of poly-aspartic acid containing proteins [9]. Importantly, dynamics of tRNA modifications has been shown to control the stage-specific gene expression in *P. falciparum* [19].

In higher eukaryotes, N^6^-methyl adenosine (m6A) modifications of mRNA and lncRNAs regulate the RNA tertiary structure and recruit the m6A binding proteins [21]. m6A is a highly conserved eukaryotic modification on DNA and RNA. However, the functions of mRNA adenosine methylation are less understood in many organisms, particularly in apicomplexan parasites such as *P. falciparum*. Recently, m6A has been identified as predominant modification on mRNAs of P. falciparum and that this post-transcriptional modification is dynamically distributed [22].

In this study, we aimed to identify and characterize the potential m6A modification methyl-reader proteins in *P. falciparum*. We searched YTH-specific homologue in the genome of the parasite and identified two putative proteins in *P. falciparum* (PfYTH), in concordance with a previous report [22]. As PfYTH2 showed higher similarity with other known eukaryotic YTH proteins, here we functionally characterized this putative methyl-binding protein. For this, we developed a modified MeRIP assay with PfYTH2 and identified that it binds strongly to m6A containing mRNA and RNA oligos in vitro. Importantly, PfYTH2 forms the m6A binding pocket with a cluster of aromatic amino acids that preferentially binds to m6A containing mRNA with high affinity. Experimental site-directed mutagenesis followed by molecular dynamics simulation analysis revealed reduced binding of PfYTH2 mutant protein to m6A-containing RNA. Next, we developed a modified methylated RNA immunoprecipitation (MeRIP) assay using recombinant PfYTH2 protein. We used this assay, along with NGS sequencing to identify the m6A specificity of PfYTH2. Interestingly, the PfYTH2 protein has more permissive sequence specificity on m6A containing mRNA than the YTH proteins of other organisms. Such permissive binding has a potential role in the regulation of selective/delayed translation of specific transcripts in *P. falciparum* during its development in RBCs. We anticipate that the findings presented here will propel further research in elucidating the role(s) of m6A on *P. falciparum* mRNAs and PfYTH proteins in regulating the development and pathogenicity of the parasite.

## Results

### Identification and biochemical characterization of PfYTH2 protein

**Fig. 1:**
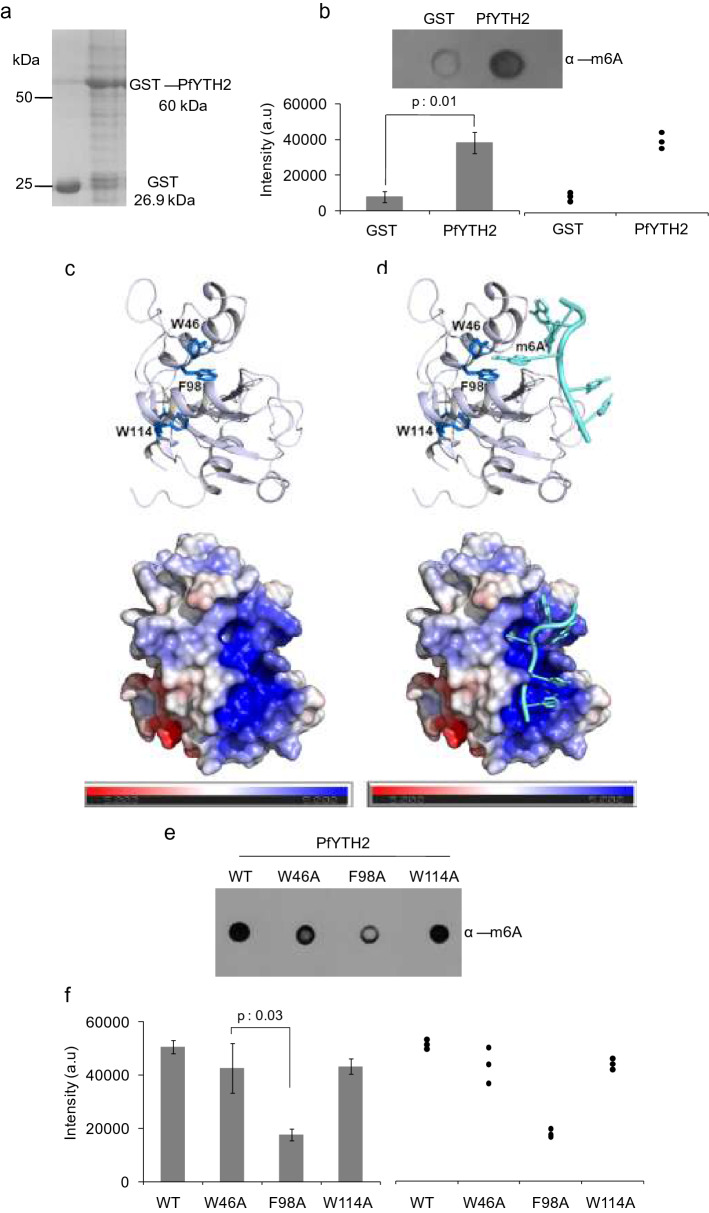
Identification and characterization of PfYTH2 protein. **a** Purification of PfYTH2 protein and normalization with GST control. **b** A representative dot blot image for the MeRIP enriched transcripts spotted on membrane. The bar plot shows intensity of spots and error bar represents SEM of three biological replicate samples. The dot plot graph on the right side represents the distribution of data points from same three independent experiments. **c** Homology model for PfYTH2 is generated and methyl-binding pocket is marked with aromatic amino acids which are shown in blue color (W46, F98 and W114). The bottom image represents the surface potential analysis that shows basic amino acids patches in blue color. **d** Superimposed structure of PfYTH2 with m6A-containing RNA ligand shows the insertion of m6A to methyl-binding pocket. The bottom image is surface potential analysis highlighting the basic patches on PfYTH2 that forms interaction surface to negatively charged m6A-containing RNA ligand. **e** A representative dot blot image for MeRIP enriched transcripts with PfYTH2 wild type (WT) and mutant proteins and probed with anti-m6A antibody. **f** The bar plot represents the intensity of each spot from Fig. 2e and the error bar represents SEM of three biological replicate samples. The dot blot graph on the right side represents the distribution of data points from same three independent experiments and the *p*-values were estimated using paired *t*-test

*Plasmodium falciparum* exhibits a high translational shift in the RBC stages during its development [3], but the regulators that facilitate these translational shifts, at the post-transcriptional level are unknown. As the *P. falciparum* mRNAs are enriched with m6A modifications, we searched for the m6A reader protein YTH homologs in the genome of *P. falciparum.* The YTH domain family proteins are known to bind m6A on mRNA in other eukaryotes [23, 24]. We performed sequence analysis and found that the *P. falciparum* genome encoded two YTH domain-containing proteins (PfYTH1 and PfYTH2), as previously reported by Baumgarten et al. [22]. Importantly, we found that PfYTH2 protein contains conserved aromatic amino acids that are essential to bind methylated adenosine (Additional file 1: Figure S1). We then cloned the PfYTH2 domain protein, expressed and purified as GST tagged protein (Additional file 1: Figure S2A–C and Fig. 1a). Next, we tested if PfYTH2 protein binds to *P. falciparum* m6A mRNAs. For this, we developed a modified MeRIP assay using PfYTH2 protein and performed methylated RNA immunoprecipitation to identify the m6A specific interaction. ImageJ analyses showed that the quality of the PfYTH2 is nearly 80%, and sufficient to perform in vitro pull-down assays (Additional file 1: Figure S3A, B). To identify the m6A enrichment in the PfYTH2 MeRIP assay, we performed dot blot assay using RNA enriched with MeRIP and probed with anti-m6A antibody. We found strong binding of PfYTH2 protein to m6A-containing RNAs compared to negligible binding in the GST control samples (Fig. 1b). This indicates that the PfYTH2 protein has the methyl-binding ability and can bind to m6A-containing transcripts.

### PfYTH2 binds to methylated mRNA via methyl-binding pocket

To obtain mechanistic insights into the PfYTH2 interaction to m6A-containing RNA, we generated the homology model of PfYTH2 protein with human YTH as a template [23] using Swiss-Model with default parameters. We found that PfYTH2 forms a methyl-binding pocket with conserved aromatic amino acids (Fig. 1c). We superimposed the m6A-containing RNA ligand with the PfYTH2 model and found that methyl adenosine precisely fits the methyl-binding pocket surrounded with aromatic amino acids in the PfYTH2 (Fig. 1d). Calculation of the electrostatic potential for the PfYTH2 homology model using the PDB2PQR server [25] (http://nbcr-222.ucsd.edu/pdb2pqr_2.0.0/) with default parameters revealed that PfYTH2 forms basic amino acid patches to bind with the negatively charged m6A-containing RNA ligand (Fig. 1c, d). To identify the potential aromatic amino acids that mediate interaction to m6A, we selected aromatic amino acids W46, F98, W114 from the methyl-binding pocket and mutated them to alanine by site-directed mutagenesis and confirmed the mutation by DNA sequencing (Additional file 1: Figures S2D and S4A). We purified the mutant proteins of similar quality (Additional file 1: Figure S4B) and tested their m6A specific interaction with RNA using modified MeRIP assay. We observed a significant reduction in binding to m6A containing mRNA for PfYTH2 F98A mutant protein, and a slight reduction in W46A mutant, whereas the W114A mutant did not show any significant loss of binding (Fig. 1e, f). Collectively, these results indicate that the *P. falciparum* PfYTH2 protein contains a functional methyl-binding pocket, which is essential to interact with m6A containing mRNA, with a possible role in regulating the mRNA functions in different stages of the parasite development in RBCs.

### Molecular dynamics simulations highlight that F98 residue is important for PfYTH2 binding to m6A containing mRNA

**Fig. 2:**
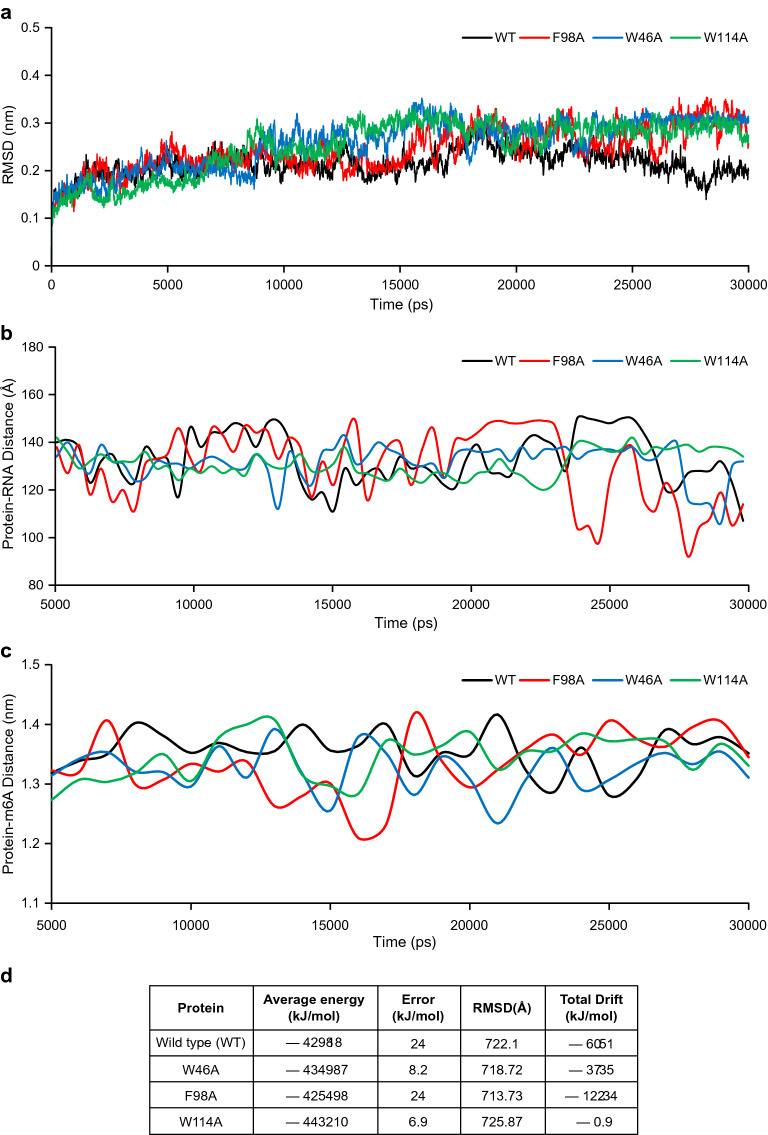
Molecular dynamics simulation analysis of wild type and mutant PfYTH2 proteins in complex with RNA. **a** Backbone Root Mean Square Deviation (RMSD) profiles of PfYTH2 over molecular dynamics simulations time scale (picoseconds). **b** Spatial distance fluctuations (Angstroms), monitored over the MD simulations timescale (picoseconds) for Wild type and mutant PfYTH proteins in complex with RNA. **c** Distance drifts computed between PfYTH wild type and mutant proteins with m6A residue from RNA (nanometers), monitored over the MD simulations timescale (picoseconds). **d** The table presents the calculated PfYTH (wild type and mutants)–RNA complex potential energy values over the molecular dynamics simulation time scale

To consolidate the m6A-specific interaction of PfYTH2 protein, we performed MeRIP pull-down assay with PfYTH2 F98A methyl-binding pocket mutant and found a significant loss of interaction with m6A containing mRNA (Fig. 1e, f). To further rationalize our observations, PfYTH2 point mutations (W46A, F98A, and W114A) were computationally designed by considering initial protein coordinates from modeled PfYTH2 structure using rosetta software package suite (https://www.rosettacommons.org/). To study the effect of the point mutations on RNA binding, we performed atomic-level molecular dynamics simulations of the mutant protein–RNA complexes for 30 ns using Gromacs simulation package [26, 27]. However, based on backbone root mean square deviation (RMSD) observations further analyses of protein–RNA complex simulations were restricted to 5–30 ns time scale to minimize the pre-equilibration artifacts (Fig. 2a).

We first assessed the overall potential energy changes of the protein–RNA complex to comprehend the effect of point mutations. Potential energies were calculated for wild type (WT) and mutant protein–RNA complexes over the simulation timescale. Interestingly, protein–RNA complexes with F98A point mutation showed a significant drift of − 122.349 (kJ/mol) over the timescale. This suggests a reduced binding interaction between the mutant PfYTH2 F98A and m6A-mRNA, corroborating well with our modified MeRIP assay, where a significant loss in binding is observed between mutant protein F98A and m6A-containing mRNA (Figs. 1e, 2b–d). In line with this observation, the average distance fluctuations monitored between F98A mutant protein and RNA also revealed significant variations compared to wild type, W46A and W114A mutations, over the simulated time scale (Fig. 2b). In addition, a higher resolution assessment between m6A residue and active site residues F98A and W46A from protein–RNA complexes revealed the minute drifting fluctuations of m6A from the residues F98A and W46A inside the active pocket compared to wild type and W114A point mutation (Fig. 2c, d). Collectively, these results highlight the importance of F98 and a possible role of W46 residues of PfYTH2 in binding with m6A mRNA.

### PfYTH2 is the m6A-specific methyl-reader protein of *P. falciparum*

**Fig. 3:**
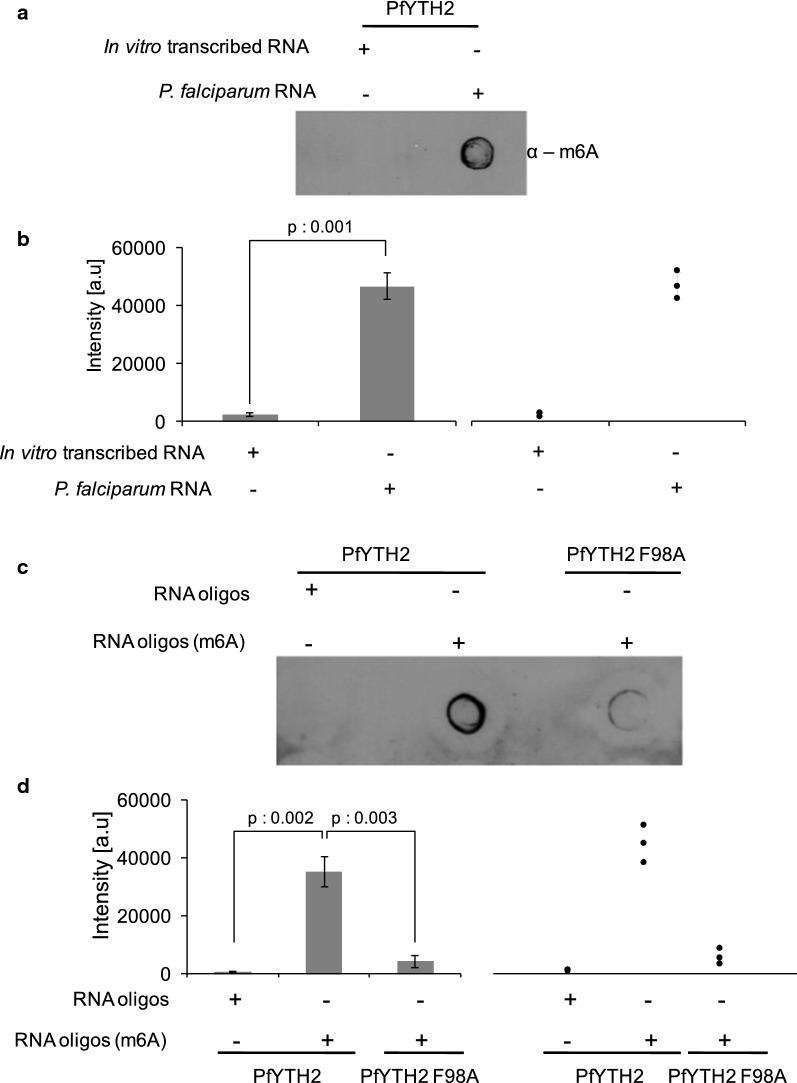
PfYTH2 specifically binds to m6A-containing RNA. **a** A representative dot blot image for the transcripts from MeRIP assay with in vitro transcribed RNA (negative control) and Pf RNA (positive control). The eluted fractions were spotted on membrane and probed with anti-m6A antibody. **b** The bar plot shows the intensity of spots and the distribution of data points from three independent experiments and error bar represents SEM of three biological replicate samples. The dot plot graph on the right side represents the distribution of data points from same three independent experiments. **c** A representative dot blot image for the transcripts from MeRIP assay with synthetic RNA oligos (positive control) with m6A modification and unmodified RNA oligos (negative control). The MeRIP assay with PfYTH2 F98A methyl-binding pocket mutant shows reduced interaction. The eluted fractions were spotted on membrane and probed with anti-m6A antibody. **d** The bar plot shows intensity of spots from three independent experiments and error bar represents SEM of three biological replicates. The dot plot graph on the right side represents the distribution of data points from same three independent experiments. Statistical significance was assessed using paired *t*-test

We next tested if PfYTH2 protein interacted with mRNAs in an m6A-dependent manner. To validate the m6A specificity of PfYTH2 protein, we adopted a two-pronged approach. First, we performed PfYTH2 pull-down experiments with in vitro transcribed RNA as control and Pf RNA as a test (Additional file 1: Figure S5A). We found a stronger interaction of PfYTH2 to Pf RNA than in vitro transcribed RNA (Fig. 3a, b). Second, we used synthetic RNA oligos containing m6A modification as a test and unmodified RNA oligos as control (Additional file 1: Figure S5B). We performed MeRIP pull-down assay for PfYTH2 protein, and dot blot assay was performed to confirm the m6A RNA oligos enrichment. We observed that PfYTH2 binds strongly to the m6A-containing RNA oligos than the unmodified RNA oligos (Fig. 3c, d).

**Fig. 4:**
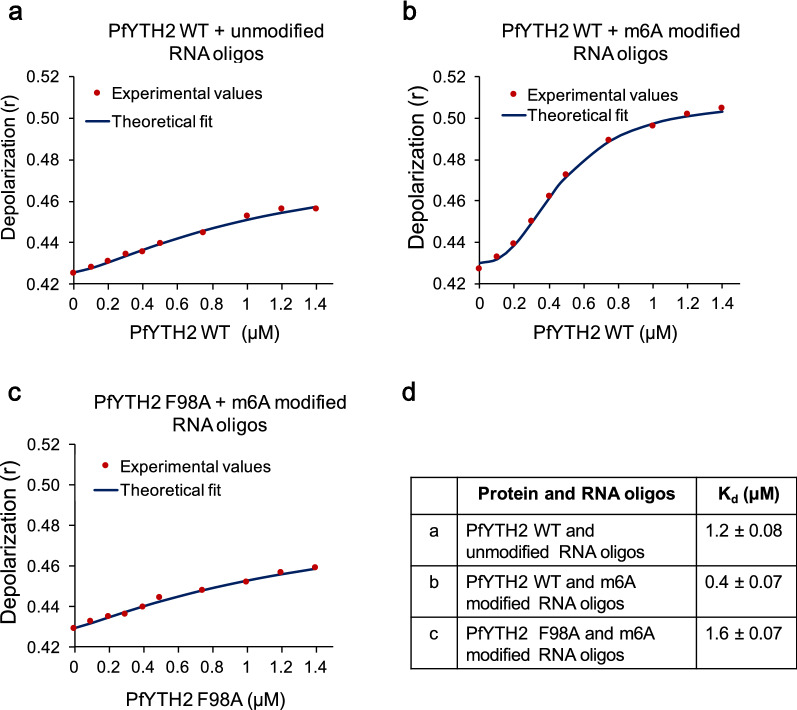
Fluorescence depolarization assay confirms the m6A specific binding of PfYTH2 protein. **a** Titration of varying concentrations of wild-type PfYTH2 protein titrated with cy5 labeled unmodified RNA oligos, followed by measurement of the depolarized fluorescence emission at different time points. The data points were fitted using Microsoft Excel. **b** Varying concentrations of wild-type PfYTH2 protein titrated with cy5 labeled m6A modified RNA oligos. **c** Various concentrations of PfYTH2 F98A mutant protein titrated with cy5 labeled m6A modified RNA oligos. Each concentration of protein variants was measured in triplicate and the average values were used to calculate the dissociation constant. **d** The calculated dissociation constant for the wild-type PfYTH2 and PfYTH2 F98A mutant protein with m6A modified and unmodified RNA oligos with standard deviation obtained from three measurements

To determine the binding affinity of PfYTH2 protein to the m6A-containing RNA, we performed fluorescence depolarization assay in solution. For this, we used cy5 fluorophore labeled m6A modified and unmodified RNA oligos. The free RNA oligos are highly depolarized with excitation of cy5 fluorophore, whereas binding of PfYTH2 protein decreases the mobility of RNA oligos and leads to increased polarization (anisotropy). To measure the binding affinity, various concentrations of PfYTH2 proteins were added to the cuvette containing m6A-containing RNA oligos. Fluorescence depolarization measurements revealed that PfYTH2 binds to m6A-containing RNA oligos with high affinity than to unmodified RNA oligos (Fig. 4a, b). The data points were fitted using a binding model and the binding constant calculation yielded *K*_d_ 0.4 ± 0.07 μM for PfYTH2 with m6A-modified RNA oligos (Fig. 4b). Based on our above experimental and MD simulation findings, we hypothesized that F98 is essential for high-affinity binding of PfYTH2 to m6A RNA. To test this, we performed the depolarization assay with PfYTH2 F98A mutant protein and found a significant loss of its binding to m6A-containing RNA oligos (Fig. 4c). The calculated dissociation constant for wild-type protein to m6A RNA oligos is four times higher affinity than the mutant F98A protein (Fig. 4d). Taken together, these results establish that PfYTH2 protein is an m6A modification reader protein, facilitated by F98 residue and may have a significant role in controlling the fate of m6A containing mRNA in *P. falciparum*.

### PfYTH2 binds to methylated mRNAs with permissive sequence selectivity

**Fig. 5:**
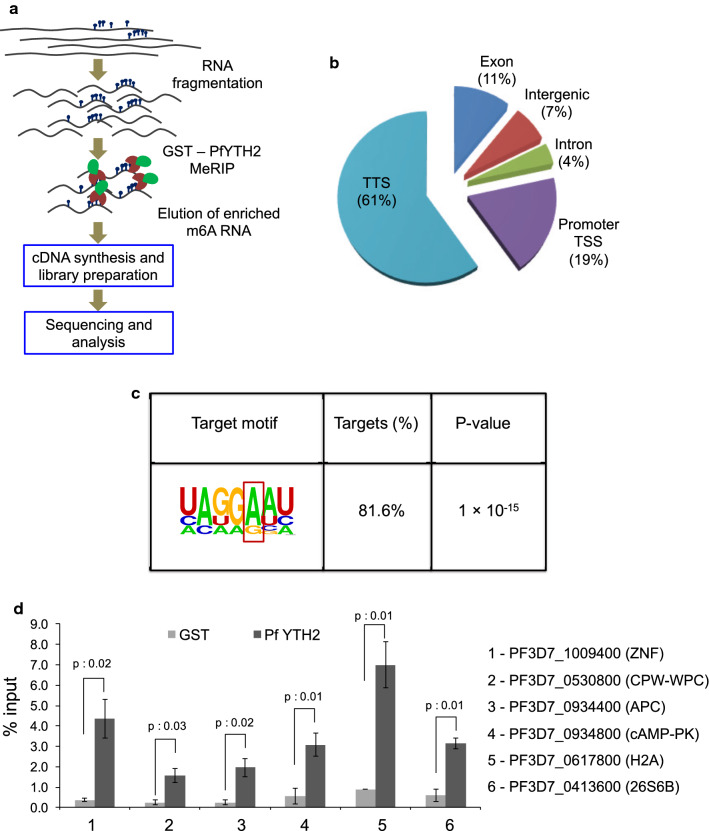
Modified MeRIP analysis using PfYTH2. **a** The experimental flow of modified MeRIP using PfYTH2. **b** Pie chart represents the PfYTH2 specific MeRIP enriched reads mapped to the genome that are normalized to GST control. More than 60% of transcripts mapped to TTS region. **c** The motif analysis of TTS specific reads enriched in PfYTH2 samples. About 80% of reads mapped to the represented motif with a significant *p*-value. **d** Validation of the PfYTH2 specific enrichment of 6 candidate transcripts by qRT-PCR analysis. PfYTH2 and GST specific enrichment was calculated to percentage input of samples, the error bar represents SEM (*n* = 3). Statistical significance was calculated using paired *t*-test

We performed a modified MeRIP assay with fragmented Pf RNA coupled with next-generation sequencing to identify m6A-containing mRNAs that are read by PfYTH2 protein (Fig. 5a, Additional file 1: Figure S6A, B). We next prepared the library from the enriched mRNA (Additional file 1: Figure S7) and sequenced using Illumina NextSeq Single-end sequencing with 75Χ1, and obtained an average of 24.16 million reads. More than 80% of the sequencing reads were successfully aligned to the *P. falciparum* genome, using Bowtie2 (Additional file 1: Figure S8). The PfYTH2 specific pull-down sample was normalized to the input control. Next, we investigated for the PfYTH2-specific enrichment of aligned reads to the genome and found that more than 60% of aligned reads corresponds to the transcription termination site (TTS), at the 3′ end of the gene (Fig. 5b). The PfYTH2-specific enrichment of sequence reads to the TTS region suggests that m6A methyl marks in *P. falciparum* are highly enriched at the 3′ end of the transcript. Notably, some of the PfYTH2-specific transcripts that are mapped to TTS regions are transcription factors and metabolism-associated genes (Table 1). This explains that expression of transcription factors and metabolic pathway proteins could be tightly regulated through PfYTH2-m6A modification specific interaction on mRNA of *P. falciparum*.

**Table 1:** Enriched transcripts in PfYTH2 pull-down samples that are mapped to TTS region of the genes

PlasmoDB identifier	Gene name
PF3D7_0317100	6-cysteine protein
PF3D7_0803500	AAA family ATPase
PF3D7_1246200	Actin I
PF3D7_0405200	ag-1 blood stage membrane protein homologue
PF3D7_0934400	AP2 domain transcription factor, putative
PF3D7_0613400	Apicoplast ribosomal protein L18 precursor, putative
PF3D7_1235700	ATP synthase subunit beta
PF3D7_1434200	Calmodulin
PF3D7_0934800	cAMP-dependent protein kinase catalytic subunit
PF3D7_0806200	C-mannosyltransferase
PF3D7_0808400	Coatomer subunit epsilon, putative
PF3D7_0530800	CPW-WPC family protein
PF3D7_0423800	Cysteine-rich positive antigen
PF3D7_0705400	DNA replication licensing factor MCM7
PF3D7_0511700	EKC/KEOPS complex subunit CGI120
PF3D7_1116500	Folate transporter 2
PF3D7_1462800	Glyceraldehyde-3-phosphate dehydrogenase
PF3D7_0610400	Histone 3
PF3D7_0617800	Histone H2A
PF3D7_1105000	Histone H4
PF3D7_1003600	Inner membrane complex protein 1c
PF3D7_0522700	Iron-sulfur assembly protein
PF3D7_1334500	MSP7-like protein
PF3D7_0722400	Obg-like ATPase 1
PF3D7_1115600	Peptidyl-prolyl cis–trans isomerase CYP19B
PF3D7_0322000	Peptidyl-prolyl cis–trans isomerase CYP19A
PF3D7_1430200	Plasmepsin IX
PF3D7_0827900	Protein disulfide-isomerase
PF3D7_0817500	Protein kinase C inhibitor-like protein
PF3D7_0927700	Serine/threonine-protein phosphatase
PF3D7_0406200	Sexual stage-specific protein
PF3D7_1418800	Signal recognition particle RNA
PF3D7_0518200	SWIB/MDM2 domain-containing protein
PF3D7_0214000	T-complex protein 1 subunit theta
PF3D7_1104400	Thioredoxin domain-containing protein
PF3D7_1439900	Triose phosphate isomerase
PF3D7_1033900	Ubiquitin-conjugating enzyme
PF3D7_1113300	UDP-galactose transporter
PF3D7_0934500	V-type proton ATPase subunit E, putative
PF3D7_0309800	YTH domain-containing protein
PF3D7_1009400	Zinc finger protein, putative

Next, we aimed to identify sequence patterns that PfYTH2 could specifically recognize or bind to. For this, we searched for potential binding motifs that could be enriched in the PfYTH2 protein-specific TTS reads. We found a motif A(C)G(AU)G(A)**A**A(UC)U(C) with guanine or adenine at −1 position to target adenine, purines at −2 and adenine at −3 to be significantly enriched in more than 80% of the TTS reads (Fig. 5c). We validated the PfYTH2 pull-down assay experiments for the 6 candidates that are transcripts of genes associated with transcription and/or metabolism, followed by qRT-PCR analysis. We found a statistically significant enrichment of the PfYTH2-specific interaction of m6A containing transcripts compared to the GST control (Fig. 5d). The structural and biophysical data of YTH proteins from higher eukaryotes show that YTH protein strongly prefers the guanine base at −1 and −2 positions to target adenine and cytosine base at +1 position and disfavor the adenine at these positions [23, 24]. The motif analyses for MeRIP sequence reads revealed that PfYTH2 has more permissible flank sequence specificity to methylated adenosine, unlike YTH proteins from higher eukaryotic organisms. This relaxation in sequence specificity could be due to the presence of only two YTH domain proteins in *P. falciparum*, as against multiple YTH domain-containing proteins in eukaryotes. Collectively, these results establish that PfYTH2 selectively binds to m6A mRNA, and has more permissive sequence specificity for binding with target methylated adenosine on mRNA of *P. falciparum*.

## Discussion

Accumulating evidences suggest that m6A modification on mRNA is a major epigenetic player that regulates the mRNA fate, translation, and stability in diverse eukaryotes [28–30]. The role of m6A modification in post-transcriptional regulation in *Plasmodium* spp. has not been studied in detail. The m6A-based regulation of mRNA is achieved through N^6^-adenosine methyltransferases (writer), adenine demethylases (erasers), and methyl-reader proteins such as the YTH family of proteins. In this study, we have identified m6A-specific methyl-binding protein PfYTH2 in *P. falciparum*, and characterized its m6A-specific binding and showed that the protein has relaxed sequence specificity to recognize the m6A containing mRNAs.

Multiple YTH domain-containing proteins are encoded by eukaryotic genome; for instance, five YTH domain-containing proteins have been identified in humans [31]. However, *P. falciparum* genome encodes only two YTH homology domain proteins [22]. Human YTH domain proteins have a stringent sequence specificity to bind with m6A containing mRNA. For instance, human YTHDC1 strictly prefers guanine at − 1 and − 2 positions to target methylated adenine [23]. Similarly, the YTH domain protein from rat prefers guanine at − 1 to target methylated adenine [32]. These findings clearly suggest that the flanking sequence of target methylated adenine is an important determinant of YTH2 binding and subsequent post-transcriptional regulation mediated by the methyl-binding proteins. Contrary to the observations made across different eukaryotic YTH2 proteins, here we identified that PfYTH2 protein has relaxed flanking sequence specificity. Importantly, Baumgarten et al., reported the presence of cytosine at +1 to the target methylated adenine in *P. falciparum* [22]. Our PfYTH2 pull-down data corroborate with this study as we observed cytosine as well as adenine and thymine nucleotides. However, the number of m6A-specific enriched transcripts identified in our PfYTH2 MeRIP is fewer than anti-m6A antibody MeRIP used by Baumgarten et al. These differences may have resulted from the differences in experimental setups as Baumgarten et al., used anti-m6A antibody for the MeRIP assay, whereas we used PfYTH2 recombinant protein to enrich the m6A-containing transcripts. Because of the high-affinity nature, the anti-m6A antibody could bind to a large number of transcripts than the recombinant protein that we used in our assay. Moreover, the binding of recombinant YTH protein to m6A-containing transcripts is influenced by flanking bases adjacent to target m6A on RNA. Nevertheless, the six candidate transcripts obtained in PfYTH2 pull-down NGS data analysis, that we validated by qRT-PCR were also identified in anti-m6A antibody MeRIP [22] (Fig. 5d). The relaxed sequence specificity of PfYTH2 might be essential to bind with diverse methylated transcripts to regulate the functions of m6A containing mRNAs in *P. falciparum*. Surprisingly, the apicomplexa group of parasites, including *P. falciparum* contains conserved writer complexes (mRNA N6-adenosine methyltransferase) [22], but does not encode a homolog for FTO and ALKBH5 proteins, well-known m6A specific demethylases in eukaryotes. The apicomplexa parasites encode the methyl-reader proteins, but the absence of adenine demethylases in their genome suggests that regulation of m6A-containing mRNA are largely controlled by YTH methyl-binding proteins in *P. falciparum*. Given these findings, it is tempting to speculate that YTH proteins may have significant functions to regulate differential stage-specific global gene expression patterns in *P. falciparum.*

## Conclusion

Apicomplexa parasites including *P. falciparum* lead a complex life cycle for multiplication to establish infection in the host(s). This parasite undergoes multiple developmental stages in different hosts and employs stringent gene regulatory mechanisms to achieve the optimal gene expression during various developmental stages. The stage-specific transcriptome and proteome analysis of *P. falciparum* have shown a delay in mRNA synthesis and its corresponding proteins between various developmental stages [3]. This suggests that strong post-transcriptional mechanisms might be involved to regulate protein synthesis in the parasite. The accumulating evidences in many eukaryotes has shown that epi-transcriptome modification on mRNAs such as m6A modification has an important role(s) in the regulation of the mRNA stability and translation. In this study, we report the m6A-specific methyl-binding activity of PfYTH2 in *P. falciparum*, and showed that the protein has relaxed sequence specificity to recognize the m6A containing mRNAs. This study sheds light on the thus far unknown layer of regulatory processes that is essential for post-transcriptional regulation in various stages of parasite development to fine-tune the stage-specific translation in *P. falciparum*.

## Methods

### *Plasmodium falciparum* 3D7 culture maintenance

The blood stage of *P. falciparum* 3D7 was cultured in the 37 °C incubator with 5% haematocrit in RPMI 1640 (Gibco Cat # 11875093) supplemented with 10% of O+ve plasma and 0.5% albumax II (Gibco Cat # 11021037) and maintained by sub-culturing when sufficient parasitemia was achieved. Growth of *P. falciparum* was verified by preparing the thin smear followed by Giemsa staining and proceeded to RNA isolation.

### Cloning, expression and purification of PfYTH2 protein

The putative YTH2-specific sequence was amplified from cDNA prepared from mRNA isolated from trophozoite culture of *P. falciparum* 3D7. The ORF sequence for each gene was cloned in pGEX6P2 (GE Healthcare) vector using restriction sites BamH1 in the forward primer and Xho1 site in the reverse primer (Additional file 1: Table S1). The PfYTH2 clone was verified by double digestion with BamHI and XhoI sites and further confirmed by DNA sequencing. The site-directed mutagenesis was performed with mutant primer by rolling circle amplification and the presence of specific mutations in the plasmids was confirmed by DNA sequencing. The expression of PfYTH2 wild type and PfYTH2 mutant proteins were carried out in BL21DE3 cells and the cells were lysed in lysis buffer (20 mM HEPES pH 7.5, 500 mM KCL, 1 mM EDTA, 1 mM DTT, 10% glycerol) using sonicator. The cell lysate was centrifuged to remove the cell debris and the supernatant passed through the GST–Sepharose beads (GE Healthcare) chromatography column and washed with lysis buffer. The GST tagged YTH proteins were eluted using elution lysis buffer containing 40 mM reduced glutathione (Sigma-Merck). The protein was subjected to dialysis process in two different dialysis buffers (dialysis buffer I—20 mM HEPES pH 7.5, 200 mM KCL, 1 mM EDTA, 1 mM DTT, 10% glycerol; dialysis buffer II—20 mM HEPES pH 7.5, 200 mM KCL, 1 mM EDTA, 1 mM DTT, 60% glycerol). The quality of the proteins was analyzed on 12% SDS-PAGE gel and stained with Coomassie brilliant blue.

### RNA isolation from *P. falciparum*

RNA was isolated from the fresh parasite pellets by lysing the parasite cells with Trizol reagent followed by Qiagen RNA isolation kit (Cat # 74104). The quality of the RNA was analyzed on formaldehyde-denatured gel and the RNA stored at − 80 °C for further experiments.

### Dot blot assay

The dot blot assay was carried out to detect the presence of methyl adenines in the immunoprecipitated RNA from MeRIP assay. The MeRIP enriched RNA was spotted on positively charged nylon membrane (GE Healthcare Cat # RPN303B) and RNA was cross-linked using UV cross-linker with 0.1 J for 6 min. Presence of RNA in the membrane was confirmed by Methylene blue staining and the stain was washed using 1X TBST, and the membrane was blocked in 5% skimmed milk overnight. The mouse anti-m6A antibody (Diagenode—Cat # C15200082) was used to probe m6A on RNA and anti-mouse HRP conjugated was used as secondary antibody. The blot was developed using chemiluminescence ECL substrate (Bio-Rad # 1705061).

### Modified methylated RNA precipitation (MeRIP) for Pf RNA

We developed modified MeRIP (methylated RNA immunoprecipitation) to study the PfYTH2 interaction with m6A-containing RNAs. The GST tagged PfYTH2 domain protein (15 µg) was coupled with GST resin. GST protein was used as a control in the parallel reaction. The protein-bound beads were washed with protein interaction buffer (50 mM, Tris pH 8.0, 100 mM KCL, 5 mM MgCl_2_, 2 mM EDTA, 0.1% Triton, 10% glycerol) for three times and a fourth wash was carried out using MeRIP buffer (50 mM Tris, pH 7.5, 100 mM NaCl, 0.05% NP40) followed by the addition of *P. falciparum* RNA and in vitro transcribed RNA for control experiments. The protein and RNA mixture was incubated overnight at 4 °C with gentle rotation. The bound RNA fractions were eluted with elution buffer containing competitor N^6^-methyladenosine (Abcam Cat # ab145715). Eluted RNA was purified using Qiagen miRNeasy purification kit (Qiagen Cat # 217004) and used for dot blot experiments. We used PfYTH protein to map the m6A modifications on the RNA of *P. falciparum*. The RNA was fragmented into ~ 300 bases at 95 °C for 2 min using fragmentation buffer (100 mM Tris pH 8, 2 mM MgCl_2_). The fragmented RNAs were subjected to interaction with PfYTH2 and GST protein (control), the complexes were incubated at 4 °C for overnight with rotation. The unbound RNAs were washed with wash buffer and the bound RNA fraction eluted with elution buffer containing N^6^-methyladenosine (Abcam Cat # ab145715). Eluted RNA was purified using Qiagen miRNeasy purification kit (Qiagen Cat # 217004) and stored at − 80 °C for sequencing.

### Fluorescence depolarization assay

We performed fluorescence depolarization assay to calculate the binding affinity of PfYTH2 with m6A-containing RNA oligos using fluorescence spectrophotometer. The assay was performed with 50 nM of m6A modified and unmodified RNA oligos (Additional file 1: Figure S5B) labeled with cy5 fluorophore by titrating with varying concentrations of PfYTH2 wild type and F98A mutation proteins. The reactions were carried out in binding buffer containing 50 mM Tris pH 7.5, 100 mM NaCl, 1 mM EDTA and 0.05% NP40. The protein–RNA interaction was studied using fluorescence depolarization with the excitation wavelength at 633 nm (band-width 10 nm) and the emission wavelength at 665 nm (band-width 10 nm). The interaction reaction was initiated with 50 nM of Cy5-labeled RNA oligos in binding buffer and increasing concentration of proteins were added for each measurement. The measurements were collected for each concentration of proteins after incubation of protein and RNA complexes at room temperature for 3 min. Each protein concentration was measured in triplicate and the average values were taken for the analysis. The data were fitted to a binary binding equilibrium to determine the equilibrium binding constant using the Microsoft excel solver module.

### MeRIP assay for synthetic RNA oligos containing m6A modification

We used methylated RNA immunoprecipitation (MeRIP) to study the m6A specificity for PfYTH2 in vitro using synthetic RNA oligos. We procured synthetic RNA oligos containing m6A with appropriate flanks and unmodified oligos for control experiments (Eurogentec, Belgium). The RNA oligos were reconstituted in RNAse-free water and the presence of m6A was verified by dot blot assay (Additional file 1: Figure S5B). We used 4 µg of RNA oligos to each modified and unmodified reaction and performed pull-down assay with PfYTH2 wild type and F98A mutant proteins. The bound RNA oligos were eluted as described above using competitor molecule. The specific enrichment of the interaction of the RNA oligos to the proteins was studied using dot blot assay. The pull-down experiment was repeated thrice and standard error of mean (SEM) was calculated. The *p*-values were estimated using paired *t* test to assess the significance of enrichment of m6A-containing RNA oligos with YTH2 in MeRIP assay.

### Computational design of mutant PfYTH2

PfYTH2 point mutation models (W46A, F98A, and W114A) for molecular dynamics simulations were computationally designed for modeled PfYTH2 using rosetta backrub method for flexible backbone design described [33]. A total of 100 models were constructed for each point mutation design, the best models were selected based on the rosetta score obtained using rosetta ref2015 scoring function [34, 35] by calculating the energy of all atomic interactions in the protein.

### Molecular dynamics simulations

The all-atom molecular dynamics simulations were carried out using GROMACS 2019.3 molecular dynamics simulation package [26, 27]. The additive Charm36 force field files were obtained for Gromacs from a previous study [36] to describe the protein–RNA complex with modified N^6^-methyl adenosine base. The protein–RNA complex was solvated by TIP3P water model in a cubic box with a distance cutoff 10 Å between the edge of the periodic box and surface of the protein–RNA complex.

The system was neutralized with Na^+^ counter ions followed by energy minimization using steepest-descent algorithm down to a 1000 kJ/mol/nm till the energy gets converged. Before production run, all systems were equilibrated for pressure and temperature by position restraining the protein–RNA for 100 ps using canonical Number of particles (N), Pressure (P) Temperature (T) and Number of particles (N), Volume (V), Temperature (T) ensembles at respective temperatures. The long-range electrostatic interactions were calculated using Particle Mesh Ewald (PME) [37] method with a grid spacing of 0.16 nm and a cutoff of 1.0 nm was used for short-range electrostatic and van der Waals interactions. Bond lengths were constrained using LINCS algorithm [38]. All simulations were performed using a 2-fs integration time step, with a coupling coefficient of tT − 0.1 ps using modified Berendsen thermostat [39], and Parrinello–Rahman pressure-coupling at 1 bar with a coupling coefficient of tP = 1 ps. All the results were analyzed using Gromacs 2019.3 package.

### Library preparation and sequencing

DNA libraries were prepared with illumine compatible SMARTer Stranded Total RNA-Seq Kit v2 (Takara Bio USA, Inc. Cat # 634411). 400 pg of total RNA was taken for first strand synthesis followed by second strand synthesis by template switching mechanism. This was further processed with addition of Illumina adaptors and barcode as per SMARTer Stranded Total RNA-Seq Kitv2 in final PCR amplification of 16 cycles. The bar code ligated libraries were purified using HighPrep beads (Cat # AC-60050) followed by library quality control check. Illumina-compatible sequencing library was quantified by Qubit fluorometer (Thermo Scientific, MA, USA) and the fragment size distribution was analyzed on Agilent 2200 Tapestation. We got Illumina-compatible sequencing libraries with mean fragment size of ~ 338 bp.

### MeRIP sequencing data analysis

The generated raw data was checked for the quality using FastQC software and reads were preprocessed to remove the adapter sequences and removal of the low quality bases (< q30) towards 3′-end using TrimGalore. We downloaded the reference genome *P. falciparum* 3D7 from PlasmoDB (https://plasmodb.org/common/downloads/release-24/Pfalciparum3D7/) and mapped the sequencing reads using Bowtie2 v0.9.6 tool with default parameters (Additional file 1: Figure S8). The transcript abundance was estimated using HTSeq-count v0.6.1. The MACS2 v2.1.4 software was used to identify peaks in GST and YTH sample with respect to input sample. MACS2 function was used to call peaks from alignment results. The input for this command includes the alignment files for IP and the control (input) samples along with the *q* value (minimum FDR) cutoff to call significant regions. The enriched peaks with *q*-value 0.01 obtained using MACS2 and fold enrichment for each peak summit was calculated against random Poisson distribution with local lambda. The MACS tool was used for peak identification, during the peak calling step. The location with the highest fragment pileup is referred as the absolute summit which provides the details about precise binding location. Further, the precise m6A position was used for HOMER annotation for the details of annotated regions. The summit file had only one base (m6A) information identified by MACS which was used as input for HOMER annotation. The HOMER annotation includes peaks in Transcription Start Site (TSS; the region that spans from core promoter and 5′ UTR), Transcription Termination Site (TTS; the region present at 3′ UTR, which could be a single nucleotide or could be short region averaged over a small population of RNA molecules), Exon (Coding), Intronic, or Intergenic. To identify the target motif and annotate transcripts from MeRIP analysis, we used HOMER software. The HOMER helps in motif identification, considering peaks with ≥ onefold change. We identified the enriched motifs in these peak regions. For this, the broad peak sequences for both samples are fetched out and analyzed for possible motifs. With total number of target sequences, the HOMER identified 120 (81.6%) number of target sequences are with the motif. It uses default the find MotifsGenome program and performs de novo motif discovery as well as checks for the enrichment of motifs. Further, the peaks are annotated using reference genome annotation file by annotate peaks program in HOMER tool with default parameter. This program first determines the distance to the nearest TTS and assigns the peak to that gene and subsequently determines the genomic annotation of the region occupied by the center of the peak/region.

### qRT-PCR analysis of PfYTH2 MeRIP enriched transcripts

To validate the NGS data of PfYTH2 pull-down samples, we performed MeRIP using PfYTH2 and GST as control for *P. falciparum* RNA. The bound RNA from PfYTH2 protein was eluted using elution buffer containing competitor (Abcam Cat # ab145715) for m6A modification. The eluted RNA was used for cDNA synthesis. We performed a cDNA synthesis using Maxima H minus First strand synthesis kit (Thermo Scientific Cat # K1681). Briefly, the eluted RNA from YTH, GST and the input RNA were subjected to DNase digestion with 10× DNase buffer for 2 min at 37 °C in the preheated water bath separately. Then, oligo dT primers, random hexamer and 10 mM dNTPs were added to the DNase treated tubes and reaction volume was made up to 15 µl. The reaction mixtures were mixed gently and incubated at 65 °C for 5 min. Then 5× reverse transcriptase buffer, Maxima H Minus enzyme mix were added and volume of the mixtures were made up to 20 µl. Afterwards, cDNA synthesis for all the reactions were carried out in PCR machine using following conditions, 25 °C for 10 min followed by 50 °C for 15 min, reactions were terminated at 85 °C for 5 min. The qRT-PCR was carried out with freshly synthesized cDNA for 6 transcripts using Sybr Green mix from Thermo Scientific was used for amplification, and in parallel we have used 1% input for qRT-PCR reaction. The enrichment of the transcripts from YTH and GST samples were calculated from the Ct values by converting the input Ct values to 100% and fold enrichment of transcripts was represented to % input.

## Supplementary information

**Additional file 1.** Additional figures and table.

## Data Availability

Not applicable.
