# Correction to: N^6^‑Adenosine methylation on mRNA is recognized by YTH2 domain protein of human malaria parasite *Plasmodium falciparum*

**DOI:** 10.1186/s13072-020-00357-5

**Published:** 2020-09-22

**Authors:** Gayathri Govindaraju, Rajashekar Varma Kadumuri, Devadathan Valiyamangalath Sethumadhavan, C. A. Jabeena, Sreenivas Chavali, Arumugam Rajavelu

**Affiliations:** 1 Pathogen Biology, Rajiv Gandhi Centre for Biotechnology (RGCB), Thycaud PO, Thiruvananthapuram, Kerala 695014 India; 2 Department of Biology, Indian Institute of Science Education and Research (IISER) Tirupati, Karakambadi Road, Tirupati, Andhra Pradesh 517507 India; 3 Manipal Academy of Higher Education, Tiger Circle Road, Madhav Nagar, Manipal, Karnataka 576104 India

## Correction to: Epigenetics & Chromatin (2020) 13:33 10.1186/s13072-020-00355-7

The original version of this article [1], unfortunately contained a mistake. The presentation of Fig. 2 has been published incorrectly. The correct Fig. 2 is provided.

**Fig. 2:**
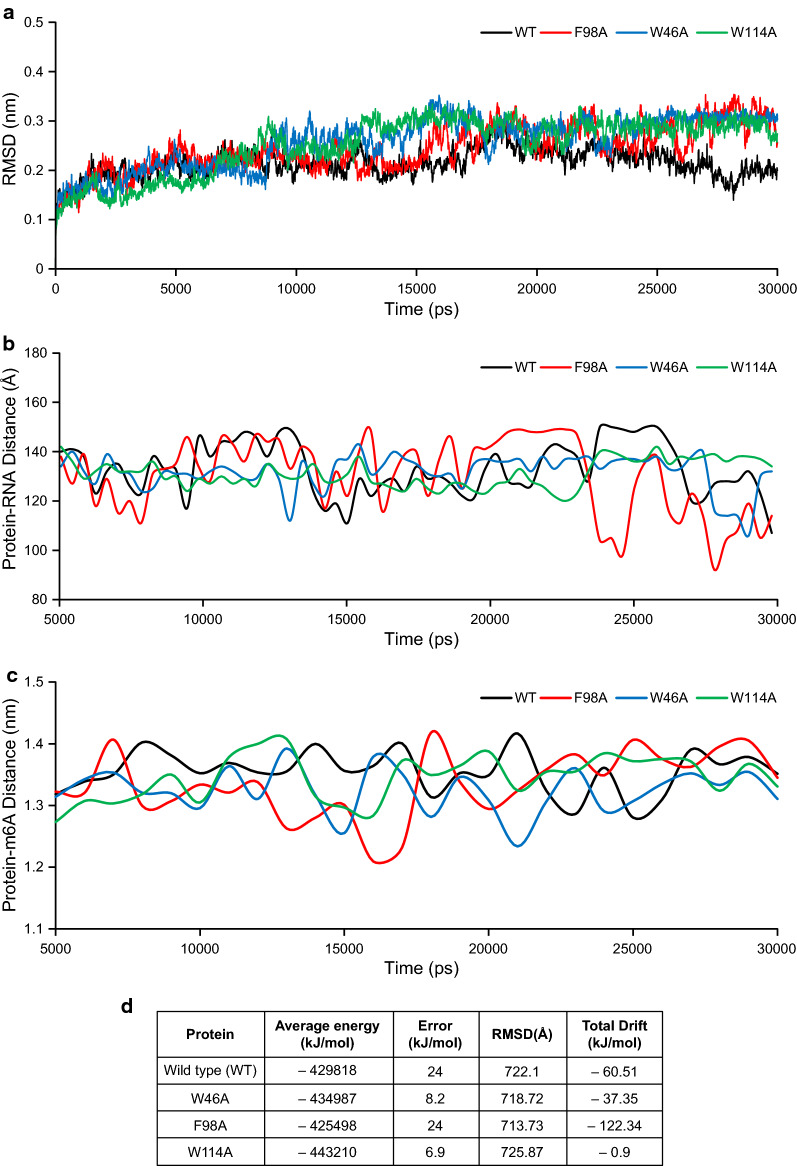
Molecular dynamics simulation analysis of wild type and mutant PfYTH2 proteins in complex with RNA. **a** Backbone Root Mean Square Deviation (RMSD) profiles of PfYTH2 over molecular dynamics simulations time scale (picoseconds). **b** Spatial distance fluctuations (Angstroms), monitored over the MD simulations timescale (picoseconds) for Wild type and mutant PfYTH proteins in complex with RNA. **c** Distance drifts computed between PfYTH wild type and mutant proteins with m6A residue from RNA (nanometers), monitored over the MD simulations timescale (picoseconds). **d** The table presents the calculated PfYTH (wild type and mutants)–RNA complex potential energy values over the molecular dynamics simulation time scale

The original article has been corrected.
