# Supplementary material for: N6-Adenosine methylation on mRNA is recognized by YTH2 domain protein of human malaria parasite Plasmodium falciparum
Source: Epigenetics Chromatin. 2020 Aug 31;13:33. doi: 10.1186/s13072-020-00355-7 (PMC7457798; doi:10.1186/s13072-020-00355-7)
Supplement: Supplementary file 1 — Additional file 1. Additional figures and table. [file 13072_2020_355_MOESM1_ESM.docx]

### Supplementary information for

### N^6^-Adenosine methylation on mRNA is recognized by YTH2 domain protein of human malaria parasite *Plasmodium falciparum*

Gayathri Govindaraju^1,3#^, Rajashekar Varma Kadumuri^2#^, Devadathan Valiyamangalath Sethumadhavan^1,3^, CA Jabeena^1,3^, Sreenivas Chavali^2^ and Arumugam Rajavelu^1^*

^1^Pathogen Biology, Rajiv Gandhi Centre for Biotechnology (RGCB), Thycaud PO, Thiruvananthapuram- 695014, Kerala, India.

^2^Department of Biology, Indian Institute of Science Education and Research (IISER) Tirupati, Karakambadi Road, Tirupati - 517507, Andhra Pradesh, India.

^3^Manipal Academy of Higher Education, Tiger Circle Road, Madhav Nagar, Manipal- 576104, Karnataka, India.

# Joint first authors

* Correspondence to be addressed: +91 471 2529598, arajavelu@rgcb.res.in

**Supplementary figure 1:** PfYTH2 protein sequence alignment with human YTH proteins confirms the conservation of the methyl-binding pocket. The amino acid positions marked with blue color boxes represent conserved aromatic amino acid residues, which were selected for site-directed mutagenesis experiments.

**Supplementary figure 2: (A)** Schematic representation of PfYTH2 as GST fused protein used in this study. (**B)** PCR amplification of PfYTH2 from cDNA synthesized from mRNA isolated from the parasite. **(C)** Double digestion with BamH1 and XhoI confirms the PfYTH2 clone in pGEX6P2. **(D)** Megaprimers synthesized with mutant primer for W46, F98 and W114 and used in rolling circle amplification to prepare mutant pGEX6P2 PfYTH2 plasmids.

**Supplementary figure 3: (A)** Image intensity analysis of PfYTH2 recombinant protein using ImageJ software. **(B)** The quality of the PfYTH2 is nearly 80%. The Y-axis in the bar plot represents the percentage of image intensity, while the x-axis represents various proteins in PfYTH2 preparation, seen in panel A.

**Supplementary figure 4: (A)** Amino acid sequence profiles of the wild type and different mutants PfYTH2 proteins **(B)** Purification of PfYTH2 mutant proteins and Coomassie staining confirms the quality and normalized concentration of the proteins that were used in MeRIP assay

**Supplementary figure 5: (A)** Dot blot assay confirms the presence of m6A on Pf RNA and absent in the *in vitro* transcribed RNA. **(B)** Dot blot assay for synthetic RNA oligos that contains the m6A modification and absence of the signal in the unmodified RNA oligos. The table below the dot blot presents the synthetic RNA oligos that were used in this study and modified adenine base is highlighted in bold and underlined.

**Supplementary figure 6: (A)** Quality of total RNA isolated from *P. falciparum* 3D7 culture and optimization RNA fragmentation that was used for MeRIP assay. **(B)** The modified MeRIP samples were loaded on denatured gel and faint band was observed with lane 7 (PfYTH2 specific pull down) and used for library preparation. The staining signal in the eluted samples was less, due to the weak input signal.

**Supplementary figure 7:** Library preparation from PfYTH2 and GST samples, with an average fragment size of 350 bp.


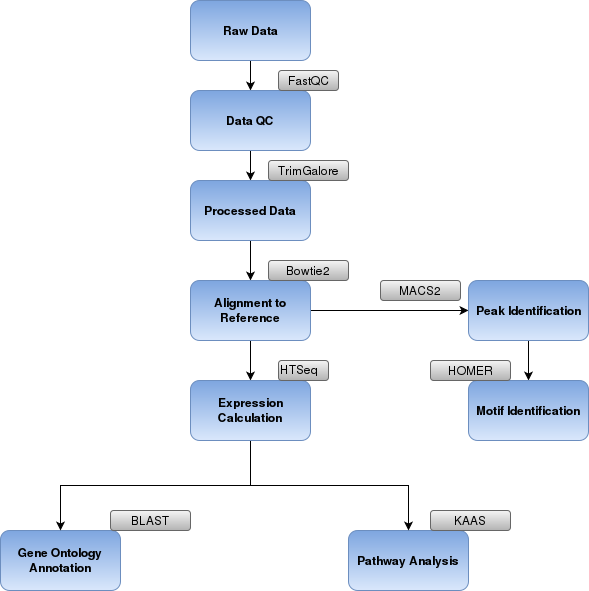


**Supplementary figure 8:** Bioinformatics analysis pipeline that was used for the analysis of MeRIP-NGS samples.

**Supplementary table 1:** List of primers used in this study.


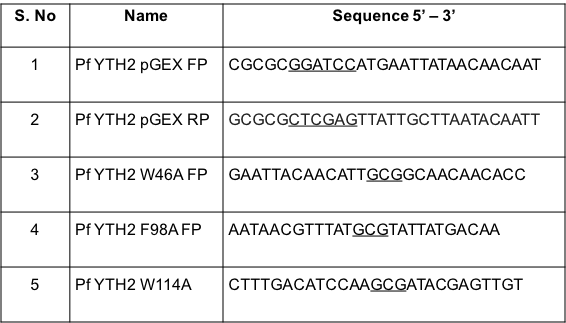


The first two primers are PfYTH2 cloning primers and the underlined regions represent the restriction sites that were used for clone the ORF into pGEX6P2 vector. The last three primers were used to generate PfYTH2 methyl-binding pocket mutants, with the underlined regions highlighting the mutated bases.
